# Multiple imputation of missing data in multilevel models with the R package mdmb: a flexible sequential modeling approach

**DOI:** 10.3758/s13428-020-01530-0

**Published:** 2021-05-23

**Authors:** Simon Grund, Oliver Lüdtke, Alexander Robitzsch

**Affiliations:** 1 IPN - Leibniz Institute for Science and Mathematics Education, Kiel, Germany; 2 Centre for International Student Assessment, Munich, Germany

**Keywords:** Multilevel analysis, Interaction effects, Missing data, Multiple imputation

## Abstract

Multilevel models often include nonlinear effects, such as random slopes or interaction effects. The estimation of these models can be difficult when the underlying variables contain missing data. Although several methods for handling missing data such as multiple imputation (MI) can be used with multilevel data, conventional methods for multilevel MI often do not properly take the nonlinear associations between the variables into account. In the present paper, we propose a sequential modeling approach based on Bayesian estimation techniques that can be used to handle missing data in a variety of multilevel models that involve nonlinear effects. The main idea of this approach is to decompose the joint distribution of the data into several parts that correspond to the outcome and explanatory variables in the intended analysis, thus generating imputations in a manner that is compatible with the substantive analysis model. In three simulation studies, we evaluate the sequential modeling approach and compare it with conventional as well as other substantive-model-compatible approaches to multilevel MI. We implemented the sequential modeling approach in the R package mdmb and provide a worked example to illustrate its application.

Multilevel models have become one of the standard tools for analyzing clustered data (e.g., with individuals clustered within groups or repeated measurements clustered within persons; see Raudenbush & Bryk 2002; Snijders & Bosker 2012). In addition, missing data are a common problem, and multiple imputation (MI) has become one of the state-of-the-art methods for dealing with them (Enders, 2010; Schafer & Graham, 2002a). An important requirement of MI is that the imputation model must “fit” the substantive analysis model in the sense that all features of the analysis must be accommodated during MI (Meng, 1994). In the context of multilevel analysis, several studies have shown that it is particularly important for the imputation model to take the multilevel structure into account if analyses based on the imputed data are to provide unbiased results (Enders et al., 2016; Lüdtke et al., 2017).

However, recent research has also demonstrated that the treatment of missing data in multilevel analyses is still challenging if the substantive analysis model contains nonlinear terms such as interactions or polynomial effects (Grund et al., 2016, 2018b; Enders et al., 2016). Nonlinear effects are extremely common in multilevel research, for example, in models that include random slopes or cross-level interactions and allow the relations between variables to differ between clusters. For this reason, it has been recommended that so-called *substantive-model-compatible* methods be used for multilevel MI (e.g., Goldstein et al., 2014; Enders et al., 2020). These methods directly take the substantive model into account during MI, thus ensuring that the imputations are always in line with the intended analysis (see also Bartlett et al., 2015).

In the present article, we present a sequential modeling approach that allows for a substantive-model-compatible treatment of missing data in multilevel research (see also Erler et al., 2016, 2017). This approach was originally proposed by Ibrahim et al., (2001) and has been considered previously in the context of regression analyses with nonlinear effects (Lüdtke et al., 2020). The purpose of this article is to extend this approach to the context of multilevel analyses with random slopes and nonlinear effects (e.g., cross-level interactions). The key feature of this approach is that the joint distribution of the variables in the imputation model is decomposed into a part that represents the substantive analysis model of interest (e.g., a multilevel model with random slopes and cross-level interaction effects) and a part that represents the model for the incomplete explanatory variables. This approach allows for a flexible specification of the imputation model, making it possible to accommodate general nonlinear associations between variables, including—but not limited to—those implied by the substantive analysis model. In addition, the approach can be used with general types of multilevel data, including hierarchical data with three or more levels and cross-classified data, as well as categorical and nonnormal data.

We also developed the mdmb package (Robitzsch and Lüdtke, 2019) for the statistical software R in which the sequential modeling approach is implemented using Bayesian estimation techniques. Although the mdmb package allows for both (a) Bayesian estimation of multilevel models and (b) multilevel MI, we focus on multilevel MI, which comes with the advantage that it separates the treatment of missing data from the analysis (Carpenter & Kenward, 2013). This can be particularly advantageous because it allows using a rich imputation model with auxiliary variables, that is, variables that are used for the treatment of missing data but are not included in the analysis model. The main advantages of using auxiliary variables are that they can increase the plausibility of the assumption that the data are missing at random (MAR) and that they allow for a more efficient use of the data. The sequential modeling approach in the mdmb package allows for the flexible treatment of auxiliary variables with multilevel data.

The article is structured as follows. In the first section, we provide a motivating example for the treatment of missing data in multilevel models with nonlinear effects. In the second section, we outline the sequential modeling approach to multilevel MI and provide a brief description of its implementation in statistical software. In the third section and the ones that follow it, we present the results of three simulation studies in which we evaluated the performance of the sequential modeling approach in different applications of multilevel MI. Finally, we provide a worked example with real data and close with a discussion of other possible applications of the sequential modeling approach, such as models with latent variables or cases with nonignorable missing data.

## Multilevel models with nonlinear effects

Suppose that we are interested in a multilevel model, in which an outcome variable *y* is regressed on an explanatory variable *x* at level 1 and an explanatory variable *z* at level 2. Suppose further that *y* and *x* are related at both levels 1 and 2 and that the effect of *x* on *y* is expected to vary both at random and as a function of *z*. This corresponds to the following multilevel model. For unit *i* (*i* = 1,…,*n*_*j*_) in cluster *j* (*j* = 1,…,*J*),

1 $$  \begin{aligned} y_{ij} & = \beta_{0} + \beta_{1} (x_{ij} - \bar{x}_{\bullet j}) + \beta_{2}  \bar{x}_{\bullet j} + \beta_{3} z_{j}\\ & \quad + \beta_{4} (x_{ij} - \bar{x}_{\bullet j}) z_{j} + \beta_{5}  \bar{x}_{\bullet j} z_{j} \\ & \quad + u_{0j} + u_{1j} (x_{ij} - \bar{x}_{\bullet j}) + e_{ij}  . \end{aligned} $$

This model includes a linear effect of the individual deviations $(x_{ij}-\bar {x}_{\bullet j})$ from the cluster mean of *x* at level 1, a linear effect of the cluster means $\bar {x}_{\bullet j}$ of *x* at level 2, and a linear effect of *z*_*j*_ at level 2, as well as a cross-level interaction effect between $(x_{ij}-\bar {x}_{\bullet j})$ and *z*_*j*_ and an interaction between $\bar {x}_{\bullet j}$ and *z*_*j*_ at level 2. In addition, the model includes a random intercept *u*_0*j*_ and a random slope *u*_1*j*_ of $(x_{ij}-\bar {x}_{\bullet j})$, thus allowing the two coefficients to vary across clusters. Such a model may be used in cross-sectional research, for example, to investigate whether the relation between students’ self-efficacy and academic achievement is moderated by school-level variables (e.g., Marsh & Rowe 1996); or in longitudinal research, for example, to investigate whether the extent to which daily stress affects individuals’ well-being depends on person-level characteristics (e.g., Sliwinski et al., (2009); see also Hoffman and Rovine 2007).

More generally, multilevel models can be used to investigate nonlinear relations between variables in a variety of ways. To this end, it is convenient to represent the multilevel model in a more compact way as a multivariate model for the response vector $\mathbf {y}_j = (y_{1j}, \ldots , y_{n_j j})$ in cluster *j*:

2 $$  \begin{array}{c} \mathbf{y}_{i} \sim N(\boldsymbol\mu_{j}, \sigma^{2} \mathbf{I}_{j}) \quad \text{with} \quad \boldsymbol\mu_{j} = h(\mathbf{x}_{j}; \boldsymbol\beta_{j})  , \end{array} $$

where *h*(⋅) represents a general nonlinear function of the explanatory variables **x**_*j*_ and the (possibly random) coefficients ***β***_*j*_, *σ*^2^ is the residual variance at level 1, and **I**_*j*_ is an identity matrix with size *n*_*j*_. In the example above, $h(\mathbf {x}_j; \boldsymbol \beta _j) = \beta _{0j} \mathbf {1}_j + \beta _{1j} (\mathbf {x}_j - \bar {\mathbf {x}}_{\bullet j}) + \beta _2 \bar {\mathbf {x}}_{\bullet j} + \beta _3 \mathbf {z}_j + \beta _4 \bar {\mathbf {x}}_{\bullet j} \mathbf {z}_j + \beta _5 (\mathbf {x}_{j} - \bar {\mathbf {x}}_{\bullet j}) \mathbf {z}_j$, where **1**_*j*_ is a vector of ones, and all values at level 2 are expanded into vectors of length *n*_*j*_ (e.g., **z**_*j*_ = *z*_*j*_**1**_*j*_). In the following, we will continue to use this compact notation and refer to the substantive analysis model in more general terms by denoting the conditional distribution of *y* given **x** as *P*(**y**|**x**) and the corresponding density function as *f*(**y**|**x**;***𝜃***) with parameters ***𝜃*** = (***β***,*σ*^2^).

The treatment of missing data in multilevel analyses can be challenging, particularly when missing data occur in the explanatory variables **x** and when the substantive analysis model includes nonlinear effects. Several authors have argued that conventional methods for multilevel MI that (a) are based on the multivariate normal distribution, thus implying only linear relations between variables, or (b) use a “reversed” imputation model (Grund et al., 2016) are not well suited for this task and can lead to biased parameter estimates (e.g., Enders et al., (2020) and Grund et al., (2018b); see also Enders et al., 2018). This is because, if the substantive analysis model *f*(**y**|**x**;***𝜃***) includes nonlinear terms such as random slopes or interaction effects, then the conditional distribution of the explanatory variables **x** given *y* tends to be more complicated than those used to generate imputations in conventional methods for multilevel MI (Kim et al., 2015). To address the challenges associated with nonlinear effects, it has been suggested that substantive-model-compatible methods be used for multilevel MI. In the following, we outline one such approach that is based on sequential modeling.

## Sequential modeling approach to multilevel MI

The main way in which MI deals with missing data is by defining a joint distribution *g*(**y**,**x**;***γ***) for all variables, from which replacements for the missing data can be drawn given the observed data. For example, in single-level data with only linear associations between the variables, one popular choice for *g*(**y**,**x**;***γ***) is the multivariate normal distribution. However, when the data have a clustered structure and there are nonlinear associations between the variables, it can be challenging to specify the joint distribution directly. For this reason, the sequential modeling approach to MI specifies the joint distribution in a sequence of models, in which every variable is represented by a conditional univariate model, including a model for the outcome variable in the substantive analysis and a model for each of the explanatory variables. In doing so, the substantive analysis model (or an extension thereof) can be directly included in the imputation model, thus ensuring that imputations are always in line with the intended analysis (for a more general account of substantive-model-compatible MI, see also Ibrahim et al., (2002; Carpenter and Kenward 2013, and Bartlett et al., 2015). Specifically, the joint distribution is modeled as follows. Let **x**_*p*_ denote the *p*^th^ explanatory variable (*p* = 1,…,*P*) in **x** = (**x**_1_,…,**x**_*P*_). Then, the sequence can be written as

3 $$  g(\mathbf{y},\mathbf{x}; \boldsymbol\gamma) = g_{y} (\mathbf{y} | \mathbf{x}; \boldsymbol\gamma_{y}) \prod\limits_{p=1}^{P} g_{x_{p}} (\mathbf{x}_{p} | \mathbf{x}_{1}, \ldots, \mathbf{x}_{p-1}; \boldsymbol\gamma_{x_{p}})  , $$

where *g*_*y*_(**y**|**x**;***γ***_*y*_) represents a conditional model for the outcome variable *y* given all explanatory variables **x** with parameters ***γ***_*y*_; and $g_{x_p} (\mathbf {x}_p | \mathbf {x}_{1}, \ldots , \mathbf {x}_{p-1}; \boldsymbol \gamma _{x_p})$ is a conditional model for *x*_*p*_ with parameters $\boldsymbol \gamma _{x_p}$, given all explanatory variables placed “earlier” in the sequence. The imputations generated in this way are said to be *substantive-model-compatible* if *f*(**y**|**x**;***𝜃***) is nested within *g*_*y*_(**y**|**x**;***γ***_*y*_), that is, if the imputation model is at least as general as the substantive analysis model (Bartlett et al., 2015); see also Lüdtke et al., (2020).

Specifying the imputation model as a sequence such as this has several advantages. For example, the conditional models can include different types of (generalized) linear mixed-effects models, which allows addressing different types of variables (e.g., discrete and continuous variables; see Lee and Mitra 2016) as well as nonnormal (e.g., skewed) data. Furthermore, the conditional models for the explanatory variables, $g_{x_p}(\mathbf {x}_p | \mathbf {x}, \ldots , \mathbf {x}_{p-1}; \boldsymbol \gamma _{x_p})$, can themselves be nonlinear, allowing for a more flexible specification of the imputation model and improved robustness in cases in which nonlinear associations might not be restricted to the outcome model *g*_*y*_(**y**|**x**;***γ***_*y*_). Finally, the sequence of models can be extended to include measurement models for latent variables, or selection or pattern-mixture models to address cases with data that are missing *not* at random (MNAR; see also Diggle & Kenward^1994^; Molenberghs et al., 1997, and Ibrahim et al., 2001).

### Markov chain Monte Carlo algorithm

The sequential modeling approach proposed here uses Markov chain Monte Carlo (MCMC) methods to estimate the parameters of the imputation models and sample imputations for the missing data from the conditional distributions of the variables (Gelman et al., 2014). To this end, the algorithm iterates along the sequence of models, where each model in the sequence takes the form of a multilevel model for variables at level 1 or a regression model for variables at level 2. For example, the multilevel model for the outcome at level 1 is given by

4 $$ g_{y}(\mathbf{y}_{j}|\mathbf{x}_{j}; \boldsymbol\gamma_{y}) = \int p(\mathbf{y}_{j}|\mathbf{x}_{j}, \mathbf{u}_{j}; \boldsymbol\gamma_{y}) p(\mathbf{u}_{j}; \boldsymbol\gamma_{y})  \mathrm{d} \mathbf{u}_{j}  , $$

where **u**_*j*_ is the vector of random effects, which are integrated out of the density. At each iteration, the algorithm provides updated values for the model parameters and imputations for the missing data. In addition, to ensure that imputations for the missing data are compatible with the substantive analysis model, the sampling steps in the MCMC algorithm are combined with Metropolis-Hastings (MH) steps for drawing the replacements for the missing data.

In the MH steps, at each iteration *t*, the algorithm draws replacements for the missing data as follows. Let $\mathbf {z}_j^{(t)} = (\mathbf {z}_{j1}^{(t)}, \ldots , \mathbf {z}_{jQ}^{(t)})$ denote the current data on both the outcome *y* and the explanatory variables **x** in cluster *j*, where the data on the *q*^th^ variable are denoted by $\mathbf {z}_{jq}^{(t)}$ (*q* = 1,…,*Q*). Let further $\mathbf {z}_{j(-q)}^{(t)} = (\mathbf {z}_{j1}^{(t)}, \ldots , \mathbf {z}_{j(q-1)}^{(t-1)},\mathbf {z}_{j(q+1)}^{(t-1)}, \ldots , \mathbf {z}_{jQ}^{(t)})$. For each variable and each cluster with incomplete data, the MH steps are carried out by first drawing values $\mathbf {z}_{jq}^{\ast } = (z_{1jq}^{\ast }, \ldots , z_{n_jjq}^{\ast })$ from a proposal distribution $N(z_{ijq}^{(t-1)}, \tau _{z_{ijq}}^2)$ specific to each value in each cluster. Then the MH ratio is calculated as

5 $$ M_{jq} (\mathbf{z}_{jq}^{{\ast}}, \mathbf{z}_{jq}^{(t-1)}) = \frac{ L_{q}(\mathbf{z}_{jq}^{{\ast}} | \mathbf{z}_{j(-q)}^{(t)}, \boldsymbol\gamma^{(t)}) }{ L_{q}(\mathbf{z}_{jq}^{(t-1)} | \mathbf{z}_{j(-q)}^{(t)}, \boldsymbol\gamma^{(t)}) }  , $$

where $L_q(\cdot | \mathbf {z}_{j(-q)}^{(t)}, \boldsymbol \gamma ^{(t)})$ is the density of the posterior distribution given the current values for all other variables **z**_*j*(−*q*)_ and the model parameters ***γ***. The proposed value $\mathbf {z}_{jq}^{\ast }$ is accepted with probability $\min \limits (M_{jq},1)$, setting $\mathbf {z}_{jq}^{(t)} = \mathbf {z}_{jq}^{\ast }$ if accepted, and $\mathbf {z}_{jq}^{(t)} = \mathbf {z}_{jq}^{(t-1)}$ otherwise.^1^

Notice that this algorithm calculates the MH ratio at level 2, thus simultaneously accepting or rejecting proposed values for all individuals within a cluster. This is required because, if the substantive model includes the cluster means of explanatory variables (e.g., Eq. 1), then imputing individual values within a cluster would change the cluster mean, thus altering the posterior distribution of the other values in the same cluster. Therefore, imputations in the MH step must be drawn simultaneously for all individuals within a cluster if the substantive model includes cluster means or other aggregated scores at level 2 (e.g., within-cluster variances or counts).

### Other methods for substantive-model-compatible multilevel MI

The sequential modeling approach (henceforth called SMC-SM) is not the only method that has been proposed in the literature for implementing an MI approach that is compatible with a substantive analysis model (see also Murray 2018). Two similar approaches have been recommended: joint modeling (SMC-JM) and the fully conditional specification (SMC-FCS). These methods—like SMC-SM—use a factorization of the joint distribution to include the substantive analysis model directly in the imputation of missing data, but they differ in how they model the explanatory variables. In the following, we provide a brief description of these approaches and discuss their similarities and differences.

#### SMC-JM

In the SMC-JM approach, the joint distribution of the data is factorized into an outcome model that pertains to the substantive analysis model and a joint model that describes all the explanatory variables simultaneously. Specifically, with SMC-JM, the joint distribution is expressed as follows:

6 $$ g(y,\mathbf{x}; \boldsymbol\gamma) = g_{y} (y | \mathbf{x}; \boldsymbol\gamma_{y})  g_{\mathbf{x}}(\mathbf{x}; \boldsymbol\gamma_{\mathbf{x}})  , $$

where *g*_**x**_(**x**;***γ***_**x**_) is a joint model for all explanatory variables. The joint model for the explanatory variables is a multivariate multilevel model, which may also include variables at level 2 or different types of variables (e.g., continuous and categorical; see also Carpenter & Kenward 2013; Quartagno & Carpenter 2019a). One of the main advantages of SMC-JM is that only a single model needs to be specified to provide imputations for all explanatory variables with missing data. One disadvantage, however, is that the joint model for the explanatory variables typically assumes that only linear associations exist between the explanatory variables, that is, that nonlinear effects are restricted to the model for the outcome variable (see also Lüdtke et al., 2020). The SMC-JM approach is implemented in the R package jomo (Quartagno et al., 2019b).

#### SMC-FCS

In the SMC-FCS approach, the general idea is similar to that of SMC-JM. However, the joint distribution of the explanatory variables is not specified directly but is instead approximated by a sequence of conditional models more similar to SMC-SM (Bartlett et al., 2015; see also van Buuren et al. 2006). The main difference between SMC-FCS and SMC-SM is that, with SMC-FCS, the conditional models are estimated separately from each other in an iterative procedure, that is,

7 $$ g_{y} (y | \mathbf{x}; \boldsymbol\gamma_{y})  \dot{g}_{x_{p}} (x_{p} | \mathbf{x}_{-p}; \dot{\boldsymbol\gamma}_{x_{p}})  , \quad \text{for all} \quad p = 1, \ldots, P  , $$

where $\dot {g}_{x_p} (x_p | \mathbf {x}_{-p}; \dot {\boldsymbol \gamma }_{x_p})$ is an imputation model for the *p*^th^ explanatory variable, given all other explanatory variables in the data set. The imputations for missing data in *x*_*p*_ are then drawn from the conditional distribution $P(x_p|y, \mathbf {x}_{-p}) \propto g_y (y | \mathbf {x}; \boldsymbol \gamma _y) \dot {g}_{x_p} (x_p | \mathbf {x}_{-p}; \dot {\boldsymbol \gamma }_{x_p})$. The SMC-FCS approach is implemented for single-level data in the R package smcfcs (Bartlett & Keogh, 2019) and for multilevel data in the standalone software Blimp^2^ (Keller & Enders, 2019).

In the following, we present the results of three simulation studies, in which we evaluated the statistical properties of these methods in a number of settings that differed in the complexity of the substantive analysis models and in the nature of the nonlinear associations that existed between the variables. The purpose of these studies was twofold. First, we aimed to evaluate the statistical properties of the SMC-SM approach in the mdmb package. Second, we aimed to compare SMC-SM with other approaches to multilevel MI including those implemented in jomo and Blimp, which are based on SMC-JM and SMC-FCS, respectively. In study 1, we evaluated the properties of the procedures in an application with a multilevel random-coefficients model, which included random slopes and interaction effects but only “overall” (i.e., conflated) effects of the explanatory variables. In study 2, we extended the multilevel analysis model to also include centering, which allowed for separate effects of the explanatory variables at levels 1 and 2, where the random slopes and interaction effects were focused on the (unconflated) effects at level 1. Finally, in study 3, we introduced additional nonlinear associations both in the substantive analysis model and between the explanatory variables.

## Study 1

### Data generation

In study 1, we generated data for three standardized variables *x*, *y*, and *z*, where *y* is an outcome variable at level 1, *x* is an explanatory variable at level 1, and *z* is an explanatory variable at level 2. We simulated the data in multiple steps. First, the explanatory variables *x* and *z* were simulated as

8 $$ \begin{aligned} x_{ij} &= x_{j}^{L2} + x_{ij}^{L1} \\ z_{j} &= z_{j}^{L2}  , \end{aligned} $$

where the level 2 components $x_j^{L2}$ and $z_j^{L2}$ followed a bivariate normal distribution with correlation *ρ*_*xz*_, and the level 1 component $x_{ij}^{L1}$ followed a univariate normal distribution. The amount of variance in *x* at level 1 and 2 was determined by its intraclass correlation (ICC, *ρ*_*Ix*_). Then, the outcome variable *y* was simulated in accordance with the following substantive analysis model

9 $$ \begin{aligned} y_{ij} & = \beta_{0} + \beta_{1} x_{ij} + \beta_{2} z_{j} + \beta_{3} x_{ij} z_{j} + u_{0j} + u_{1j} x_{ij} + e_{ij}  . \end{aligned} $$

**Fig. 1:**
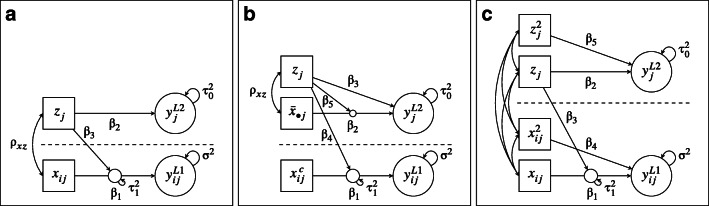
Schematic representation of the substantive analysis models in study 1 (**a**), study 2 (**b**), and study 3 (**c**). $x_{ij}^c$ = $(x_{ij} - \bar {x}_{\bullet j})$

This model is illustrated in Fig. 1 (panel A) and includes an “overall” (i.e., conflated) effect of *x* at level 1 (for a discussion, see Hoffman 2019; Preacher et al., 2016), an effect of *z* at level 2, and a cross-level interaction (CLI) between *x* and *z*. In addition, the model includes a random intercept and a random slope for the overall effect of *x*. The random effects, *u*_0*j*_ and *u*_1*j*_, and the residuals at level 1 were distributed as follows:

10 $$ \begin{array}{c} (u_{0j}, u_{1j})^{T} \sim N (\mathbf{0}, \mathbf{T} )  , \quad \mathbf{T} = \begin{bmatrix} {\tau_{0}^{2}} & \\ \tau_{01} & {\tau_{1}^{2}} \end{bmatrix}  , \\ e_{ij} \sim N(0,\sigma^{2})  . \end{array} $$

In the data generating model, we fixed the variance of the random slopes to a certain value, whereas the random intercepts and the residuals at level 1 were chosen in such a way that the ICC of *y* (*ρ*_*Iy*_) approximately matched a certain value.

#### Missing data

We induced missing data in *x* on the basis of the values in *y* by simulating a (latent) propensity for missing data using the following linear model:

11 $$ r_{ij} = \lambda_{0} + \lambda_{1} y_{ij} + v_{ij}  , \quad v_{ij} \sim N(0, 1 - {\lambda_{1}^{2}})  , $$

where *λ*_0_ is a quantile of the standard normal distribution and corresponds to the probability of missing data (e.g., − 0.674 for 25% missing data), and *λ*_1_ controls the missing data mechanism. A value in *x* was deleted if the corresponding value in *r* was larger than 0.

#### Simulated conditions

The simulated conditions are summarized in Table 1. We varied the sample sizes at level 1 (*n* = 10, 20) and level 2 (*J* = 50, 100, 200, 500, 1000) in order to study both the small- and large-sample behavior of the methods used to treat missing data. We set the ICCs to be equal for *x* and *y* and varied them to include small, moderate, and large values (*ρ*_*Ix*_ = *ρ*_*Iy*_ = .10, .20, .50), where the smaller values reflect conditions typically found in cross-sectional research, and the larger ones are more common in longitudinal research. For the regression coefficients, we chose a moderate effect of *x* (*β*_1_ = .40), a small to moderate effect of *z* at level 2 (*β*_2_ = .20), and a small to moderate CLI (*β*_3_ = .20). For the slope variance, we chose a fixed value of $\tau _1^2$ = .10, which corresponds to conditions in which the slope variance was large, moderate, or small in relation to the ICCs. Finally, we chose a fixed value of *ρ*_*xz*_ = .20 for the correlation between *x* and *z* and assumed that the random effects were uncorrelated (*τ*_01_ = 0). In the generation of missing data, we simulated data that were missing completely at random (MCAR) by setting *λ*_1_ = 0 or missing at random (MAR) by setting *λ*_1_ = .35 or .70. Finally, we set the proportion of missing data to 30%. Each simulated condition was replicated 1000 times.

**Table 1:** Simulated conditions in studies 1, 2, and 3

Design factor	Study 1	Study 2	Study 3
*Data structure*			
Level 1 sample size	10, 20	10, 20	20
Level 2 sample size	50, 100, 200, 500, 1000	50, 100, 200, 500, 1000	1000
ICCs of *X* and *Y*	.10, .20, .50	.20	.20
*Explanatory variable model*			
Correlation of *X* and *Z*	.20	.20	
Total $R_{xz}^2$			.50
Nonlinear proportion of $R^2_{xz}$			0, .25, .50, .75, 1
*Substantive analysis model*			
Effect of *x*_*ij*_	.40		.15
Effect of $(x_{ij} - \bar {x}_{\bullet j})$		.40	
Effect of $\bar {x}_{\bullet j}$		0	
Effect of *z*_*j*_	.20	.20	.15
Effect of *x*_*ij*_*z*_*j*_	.20		.15
Effect of $(x_{ij} - \bar {x}_{\bullet j}) z_j$		.20	
Effect of $\bar {x}_{\bullet j} z_j$		0	
Effect of $x_{ij}^2$			.15
Effect of $z_j^2$			.15
*Missing data*			
Proportion missing	30%	30%	30%
Effect of *Y* on missingness	0, .35, .70	0, .35, .70	0, .35, .70

Note. ICC = intraclass correlation

### Procedures

The procedures used for the treatment of missing data included both conventional and substantive-model-compatible methods for multilevel MI. Reflecting the more conventional approach to multilevel MI, we used an FCS approach based on the R package mice (van Buuren & Groothuis-Oudshoorn, 2011) in which we used a “reversed” imputation model to treat missing data in the explanatory variable with passive imputation^3^ of the interaction effect (see also Grund et al., 2016, 2018b; Enders et al., 2016). The substantive-model-compatible methods for multilevel MI included the sequential modeling approach (SMC-SM) implemented in the mdmb package as well as the SMC-JM approach implemented in jomo, and the SMC-FCS approach implemented in Blimp. All of the substantive-model-compatible methods were able to fully accommodate the model of interest, so we expected that they would perform similarly well. In addition, we also included analyses of the complete data (CD) and listwise deletion (LD) to make comparisons. Each method was carried out with an appropriate number of iterations to ensure the convergence of the procedures, which we determined by checking the convergence criteria ($\hat {R}$; Gelman & Rubin 1992) and diagnostic plots in a subset of the simulated conditions in which we expected the convergence to be slowest (e.g., small samples, small ICCs). All methods were specified with noninformative priors.^4^ Ten imputations were used throughout the simulation.

### Parameters of interest and pooling

We estimated the substantive analysis model using the R package lme4 (Bates et al., 2019), and we used Rubin’s (1987) rules to pool the parameter estimates across the imputed data sets. The parameters of interest in the substantive analysis model were primarily the regression coefficients for the effect of *x* (*β*_1_) and the CLI of *x* with *z* (*β*_3_) as well as the slope variances of the effect of *x* ($\tau _1^2$). To compare the statistical properties of the parameter estimates under each method, we calculated the bias, the root mean square error (RMSE), and the coverage rates of the 95% confidence intervals (if applicable) for each parameter of interest.

### Results

**Fig. 2:**
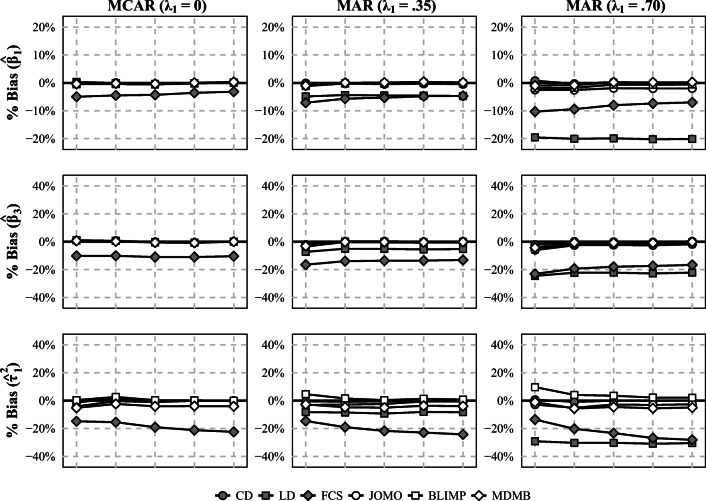
Bias (in %) of the estimated regression coefficients for the overall effect of *x* (*β*_1_), the CLI (*β*_3_), and the slope variance ($\tau _1^2$) in conditions with small samples at level 1 (*n* = 10) and moderate ICCs (*ρ*_*Ix*_ = *ρ*_*Iy*_ = .20) in study 1. *J* = level 2 sample size; CD = complete data; LD = listwise deletion; FCS = multilevel MI (fully conditional specification); JOMO = substantive-model-compatible multilevel MI (joint modeling); BLIMP = substantive-model-compatible multilevel MI (fully conditional specification); MDMB = substantive-model-compatible multilevel MI (sequential modeling). The Monte Carlo error ranged from 0.1% to 1.5% (median 0.4%)

In the interest of space, we focus on the main findings here. The full set of results is provided in Supplement B of the online supplemental materials (https://osf.io/aeqd2). The results for the bias in the estimated parameters are summarized in Fig. 2 and Table 2. In most of the simulated conditions, using conventional methods for multilevel MI (FCS) yielded biased results for the parameters of interest. This bias was largest for the CLI (*β*_3_) and the slope variance ($\tau _1^2$) but also affected the other regression coefficients (*β*_1_). Further, LD provided unbiased results only when the data were MCAR (*λ*_1_ = 0) but biased results when they were MAR (*λ*_1_ = .35, .70). By contrast, the substantive-model-compatible approaches to multilevel MI (Blimp, jomo, and mdmb) provided unbiased results for all parameters of interest in most of the simulated conditions. However, the procedures sometimes differed in how well they estimated the slope variance. Specifically, the estimated slope variance was sometimes downwardly biased with mdmb, primarily in conditions with small samples at level 1 (*n* = 10) and large ICCs (*ρ*_*Ix*_ = *ρ*_*Iy*_ = .50), whereas it was upwardly biased with Blimp, primarily in conditions with small samples at both level 1 (*n* = 10) and level 2 (*J* = 50).

**Table 2:** Bias (in %) of the estimated regression coefficient for the overall effect of *x* (*β*_1_), the CLI (*β*_3_), and the slope variance ($\tau _1^2$) in study 1

			MCAR (*λ*_1_ = 0)						MAR (*λ*_1_ = .70)					
*n*	*J*	Par.	CD	LD	FCS	JOMO	BLIMP	MDMB	CD	LD	FCS	JOMO	BLIMP	MDMB
			*ρ*_*Ix*_ = *ρ*_*Iy*_ = .10											
10	200	*β*_1_	0.1	0.1	− 4.0	0.0	− 0.1	0.1	− 0.1	**−20.5**	− 8.7	− 2.3	− 0.9	− 0.1
		*β*_3_	0.5	0.3	**−10.5**	0.3	0.2	0.2	0.4	**−21.9**	**−18.0**	− 1.8	− 0.4	0.0
		$\tau _1^2$	− 0.2	− 0.4	**−19.7**	− 1.0	0.2	− 3.7	− 0.1	**−31.3**	**−23.4**	− 3.0	2.7	− 4.2
	1000	*β*_1_	− 0.0	0.0	− 3.4	− 0.0	− 0.1	0.1	0.1	**−20.3**	− 7.0	− 1.9	− 0.4	0.2
		*β*_3_	0.1	− 0.1	**−10.8**	− 0.1	− 0.1	− 0.2	− 0.6	**−22.9**	**−17.7**	− 2.7	− 1.5	− 0.9
		$\tau _1^2$	0.1	0.4	**−22.2**	0.3	0.5	− 1.8	− 0.1	**−31.8**	**−28.3**	− 3.1	1.8	− 3.2
20	200	*β*_1_	0.6	0.6	− 3.4	0.6	0.5	0.6	− 0.1	**−19.8**	− 7.6	− 2.0	− 0.9	− 0.4
		*β*_3_	− 0.2	− 0.2	− 9.9	− 0.3	− 0.2	− 0.3	0.9	**−20.7**	**−15.2**	− 1.1	− 0.9	0.3
		$\tau _1^2$	0.1	0.0	**−19.2**	− 0.3	0.1	− 0.5	− 0.8	**−32.3**	**−25.0**	− 4.0	2.1	− 1.0
	1000	*β*_1_	− 0.2	− 0.2	− 3.9	− 0.2	− 0.2	− 0.1	− 0.0	**−19.7**	− 6.8	− 1.8	− 0.7	− 0.1
		*β*_3_	0.1	0.2	− 9.6	0.2	0.1	0.1	0.0	**−21.6**	**−15.7**	− 2.0	− 1.9	− 0.6
		$\tau _1^2$	0.1	0.1	**−20.4**	0.1	0.2	− 0.3	− 0.3	**−31.7**	**−26.6**	− 3.2	2.7	− 0.3
			*ρ*_*Ix*_ = *ρ*_*Iy*_ = .20											
10	200	*β*_1_	− 0.4	− 0.4	− 4.3	− 0.4	− 0.6	− 0.3	0.3	**−19.9**	− 8.0	− 1.9	− 0.6	0.2
		*β*_3_	− 0.1	− 0.3	**−11.0**	− 0.4	− 0.5	− 0.5	− 0.1	**−22.2**	**−17.9**	− 2.4	− 1.1	− 0.8
		$\tau _1^2$	0.2	− 0.3	**−19.1**	− 1.0	0.3	− 4.0	− 0.1	**−30.3**	**−23.3**	− 2.8	3.5	− 4.6
	1000	*β*_1_	0.2	0.1	− 3.2	0.1	0.1	0.3	0.1	**−20.1**	− 7.0	− 2.0	− 0.6	0.2
		*β*_3_	0.1	0.1	**−10.4**	0.1	0.1	0.1	0.3	**−22.1**	**−16.6**	− 1.8	− 0.5	− 0.1
		$\tau _1^2$	− 0.0	− 0.2	**−22.4**	− 0.4	− 0.1	− 4.1	0.1	**−30.5**	**−28.1**	− 2.6	2.1	− 5.0
20	200	*β*_1_	0.1	0.0	− 3.9	0.0	− 0.0	0.1	0.1	**−19.2**	− 7.5	− 1.9	− 1.1	− 0.5
		*β*_3_	0.2	0.0	− 9.4	0.1	0.2	0.1	0.7	**−20.9**	**−15.4**	− 1.7	− 1.7	− 0.7
		$\tau _1^2$	− 0.2	− 0.4	**−19.0**	− 0.6	0.1	− 1.4	− 0.3	**−30.4**	**−24.0**	− 2.6	4.4	− 0.0
	1000	*β*_1_	− 0.0	− 0.0	− 3.7	− 0.0	− 0.0	0.1	− 0.1	**−19.4**	− 7.0	− 2.0	− 1.1	− 0.4
		*β*_3_	0.1	0.1	− 9.3	0.2	0.1	0.1	− 0.2	**−21.7**	**−15.6**	− 2.3	− 2.4	− 1.3
		$\tau _1^2$	0.1	0.1	**−20.1**	0.1	0.2	− 1.0	− 0.3	**−30.4**	**−26.4**	− 2.6	3.8	− 0.8
			*ρ*_*Ix*_ = *ρ*_*Iy*_ = .50											
10	200	*β*_1_	0.5	0.5	− 3.6	0.3	0.3	0.1	0.1	**−16.1**	− 7.8	− 2.2	− 1.2	− 0.5
		*β*_3_	0.2	0.2	− 10.0	0.3	0.3	− 0.5	− 0.1	**−19.1**	**−18.1**	− 3.2	− 2.2	− 3.0
		$\tau _1^2$	− 0.3	0.0	**−18.7**	− 1.0	0.3	**−20.0**	0.1	**−23.0**	**−22.5**	− 0.2	5.6	**−25.1**
	1000	*β*_1_	0.0	− 0.1	− 3.4	− 0.0	− 0.0	− 0.1	− 0.0	**−16.2**	− 7.0	− 2.1	− 0.9	− 0.3
		*β*_3_	− 0.0	− 0.1	**−10.2**	− 0.0	− 0.1	− 0.9	0.2	**−18.5**	**−16.3**	− 2.1	− 1.1	− 2.1
		$\tau _1^2$	− 0.0	− 0.2	**−21.8**	− 0.1	0.3	**−20.5**	0.2	**−23.1**	**−27.1**	− 0.2	3.7	**−29.9**
20	200	*β*_1_	− 0.3	− 0.2	− 4.3	− 0.3	− 0.3	− 0.5	0.1	**−14.5**	− 7.3	− 2.6	− 2.1	− 3.1
		*β*_3_	− 0.4	− 0.1	− 9.5	− 0.2	− 0.2	− 0.9	− 0.3	**−17.1**	**−16.1**	− 4.1	− 5.2	− 6.9
		$\tau _1^2$	0.8	0.9	**−18.0**	0.3	1.0	− 4.2	− 0.3	**−22.2**	**−22.8**	2.0	8.7	− 3.3
	1000	*β*_1_	0.0	0.1	− 3.8	0.1	0.1	− 0.2	0.0	**−14.5**	− 6.8	− 2.4	− 1.7	− 2.7
		*β*_3_	0.1	0.1	− 9.4	0.1	0.0	− 0.6	0.3	**−16.6**	**−15.0**	− 3.0	− 3.5	− 5.3
		$\tau _1^2$	0.0	0.1	**−20.1**	− 0.0	0.1	− 5.0	0.1	**−22.1**	**−24.8**	2.0	7.3	− 4.1

Note. Biases larger than ± 10*%* are printed in bold. *n* = level 1 sample size; *J* = level 2 sample size; *ρ*_*Ix*_ = *ρ*_*Iy*_ = intraclass correlations of *x* and *y*; CD = complete data; LD = listwise deletion; FCS = multilevel MI (fully conditional specification); JOMO = substantive-model-compatible multilevel MI (joint modeling); BLIMP = substantive-model-compatible multilevel MI (fully conditional specification); MDMB = substantive-model-compatible multilevel MI (sequential modeling). The Monte Carlo error ranged from 0.1% to 0.8% (median 0.2%)

The overall accuracy of the parameter estimates in terms of the RMSE largely followed the same pattern as the bias. Specifically, the RMSE was usually smallest with Blimp, jomo, and mdmb, and the differences between these procedures were negligible despite the differences in the bias (e.g., for the slope variance). For the parameter estimates obtained with FCS, the results for the RMSE were more mixed. Specifically, as compared with the substantive-model-compatible methods for multilevel MI, the RMSE of the estimates obtained with FCS tended to be larger for regression coefficients but smaller for the slope variance. Finally, the RMSE of the estimates obtained with LD was usually larger than with the other methods, especially when the data were MAR.

The coverage rates of the 95% confidence intervals are summarized in Table 3 for the regression coefficients of the CLI (*β*_3_). For Blimp, jomo, and mdmb, the coverage rates remained close to the nominal level of 95% in most of the simulated conditions. By contrast, the coverage rates dropped well below the nominal level with FCS, especially in larger samples. Finally, with LD, the coverage rates were close to 95% when the data were MCAR but much lower when the data were MAR, especially in conditions with larger samples and with a stronger MAR mechanism (*λ*_1_ = .70).

**Table 3:** Coverage of the 95% confidence intervals for the CLI (*β*_3_) in conditions with moderate ICCs (*ρ*_*Ix*_ = *ρ*_*Iy*_ = .20) in study 1

		MCAR (*λ*_1_ = 0)						MAR (*λ*_1_ = .70)					
*n*	*J*	CD	LD	FCS	JOMO	BLIMP	MDMB	CD	LD	FCS	JOMO	BLIMP	MDMB
10	50	94.0	93.7	94.5	94.0	94.3	93.6	93.8	**85.2**	**92.4**	94.2	94.1	94.7
	100	96.0	96.0	94.2	95.3	95.8	95.1	94.1	**82.0**	**89.4**	95.1	93.7	93.5
	200	93.6	93.4	**89.9**	93.1	93.5	93.4	95.1	**66.0**	**80.9**	96.6	95.3	94.8
	500	94.6	94.7	**78.8**	94.6	94.9	94.0	94.8	**32.8**	**58.9**	97.4	94.7	94.1
	1000	94.8	95.2	**68.1**	96.1	95.4	95.5	96.4	**7.5**	**31.5**	99.1	95.6	95.3
20	50	93.5	92.9	92.6	93.3	93.6	93.1	93.8	**85.5**	**91.5**	95.4	95.1	94.1
	100	94.5	94.1	**90.8**	94.8	93.9	94.5	94.9	**75.6**	**87.2**	96.1	95.5	94.5
	200	95.2	95.9	**90.1**	96.1	96.2	95.9	93.7	**59.7**	**77.7**	95.9	93.2	93.8
	500	95.2	94.9	**79.9**	94.8	94.9	94.2	96.0	**21.5**	**50.4**	98.1	94.1	95.4
	1000	94.7	94.6	**62.5**	94.8	94.6	94.2	94.9	**2.1**	**21.1**	98.7	**91.4**	93.9

Note. Coverage rates below 92.5% are printed in bold. *n* = level 1 sample size; *J* = level 2 sample size; CD = complete data; LD = listwise deletion; FCS = multilevel MI (fully conditional specification); JOMO = substantive-model-compatible multilevel MI (joint modeling); BLIMP = substantive-model-compatible multilevel MI (fully conditional specification); MDMB = substantive-model-compatible multilevel MI (sequential modeling). The Monte Carlo error ranged from 0.2% to 1.1% (median 0.5%)

### Summary

The results of study 1 indicated that substantive-model-compatible methods for multilevel MI such as those implemented in jomo (SMC-JM), Blimp (SMC-FCS), and mdmb (SMC-SM) can all provide unbiased parameter estimates in multilevel analyses with missing data when the substantive analysis model includes random slopes or nonlinear effects. By contrast, using LD or conventional methods for multilevel MI such as FCS can have the potential to provide strongly biased estimates of the parameters in the substantive analysis model. However, in study 1, we focused on the relatively simple scenario, in which the substantive analysis model included only “overall” (i.e., conflated) effects. For this reason, in the following study, we expanded the substantive analysis model to include centering, which allows the explanatory variables to have different effects at levels 1 and 2.

## Study 2

### Data generation

In study 2, we generated the explanatory variables *x* and *z* in the same way as in study 1. However, in the substantive analysis model, which we used to generate the outcome variable *y*, this time we used cluster-mean centering to decompose *x* into two separate components, $(x_{ij} - \bar {x}_{\bullet j})$ and $(\bar {x}_{\bullet j})$, thus allowing the effect of *x* on *y* to differ between levels 1 and 2:

12 $$ \begin{aligned} y_{ij} & = \beta_{0} + \beta_{1} (x_{ij} - \bar{x}_{\bullet j}) + \beta_{2}  \bar{x}_{\bullet j} + \beta_{3} z_{j}\\ &\quad+ \beta_{4} (x_{ij} - \bar{x}_{\bullet j}) z_{j} + \beta_{5}  \bar{x}_{\bullet j} z_{j} \\ & \quad + u_{0j} + u_{1j} (x_{ij} - \bar{x}_{\bullet j}) + e_{ij}  . \end{aligned} $$

This model was already shown in the motivating example above and is also illustrated in Fig. 1 (panel B). In contrast to the model used in study 1, this model includes both the cluster means of *x* ($\bar {x}_{\bullet j}$) and the within-cluster deviations $(x_{ij} - \bar {x}_{\bullet j})$ as separate explanatory variables, thus estimating separate regression coefficients for the effects of *x* on *y* at levels 1 and 2. In addition, the model includes an effect of *z* at level 2 as well as a CLI of $(x_{ij} - \bar {x}_{\bullet j})$ with *z*_*j*_, a level 2 interaction between $\bar {x}_{\bullet j}$ and *z*_*j*_, and a random slope of $(x_{ij} - \bar {x}_{\bullet j})$. The distributions of the random effects and residuals were the same as in study 1, and missing data were induced in *x* as before.

#### Simulated conditions

The simulated conditions are summarized in Table 1. For most aspects of the design, we left the simulated conditions unchanged. For the regression coefficients, we chose a moderate effect of $(x_{ij} - \bar {x}_{\bullet j})$ at level 1 (*β*_1_ = .40), no effect of $\bar {x}_{\bullet j}$ at level 2 (*β*_2_ = 0), a small to moderate effect of *z* at level 2 ($\beta _{2}^{3}$ = .20), a small to moderate CLI (*β*_4_ = .20), and no interaction at level 2 (*β*_5_ = 0). As before, each condition was replicated 1000 times.

### Procedures and parameters of interest

The procedures used for the treatment of missing data were the same as in study 1. However, not all software implementations of the substantive-model-compatible methods for multilevel MI allowed the cluster means and the centered scores of the explanatory variables to be included in the specification of the substantive analysis model. Specifically, only the SMC-SM approach in mdmb and the SMC-FCS approach in Blimp allowed the cluster means and centered variables to be included, whereas the SMC-JM approach in jomo allowed explanatory variables to be included only “as is” (i.e., with conflated effects). In this context, it is worth noting that Blimp and mdmb handle the cluster means in slightly different ways, where mdmb includes *manifest* cluster means, whereas Blimp includes *latent* clusters means (for a detailed discussion, see Lüdtke et al., 2008). However, previous studies have found that the two options tend to produce similar results unless in extreme cases (e.g., with strongly imbalanced sample sizes across clusters; see Grund et al., 2018a; Resche-Rigon & White 2018). For this reason, both Blimp and mdmb can be considered compatible with the substantive analysis model, and we expected that they would both perform better than jomo. The parameters of interest were primarily the regression coefficients of the effect of *x* at level 1 (*β*_1_) and the CLI (*β*_4_) as well as the slope variances of the effect of *x* at level 1 ($\tau _1^2$). For each procedure and parameter of interest, we calculated the bias, the RMSE, and the coverage rates of the 95% confidence intervals as before. However, in the interest of space, we focus here on the bias and coverage rates.

### Results

**Fig. 3:**
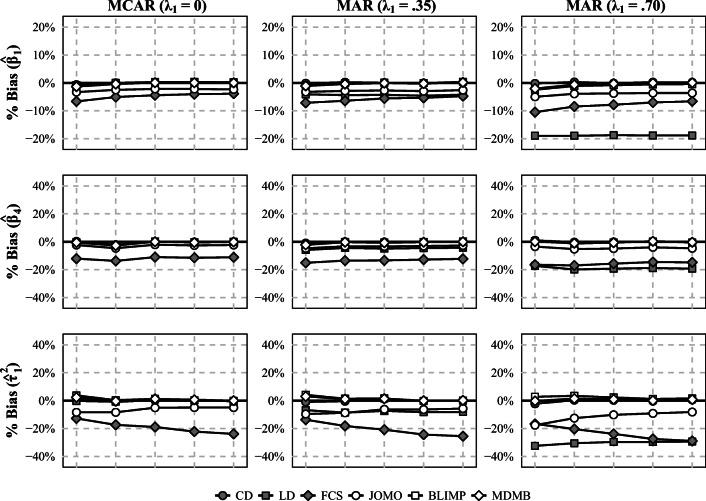
Bias (in %) of the estimated regression coefficients for the effect of *x* at level 1 (*β*_1_), the CLI (*β*_4_), and the slope variance ($\tau _1^2$) in conditions with small samples at level 1 (*n* = 10) and moderate ICCs (*ρ*_*Ix*_ = *ρ*_*Iy*_ = .20) in study 2. *J* = level 2 sample size; CD = complete data; LD = listwise deletion; FCS = multilevel MI (fully conditional specification); JOMO = substantive-model-compatible multilevel MI (joint modeling); BLIMP = substantive-model-compatible multilevel MI (fully conditional specification); MDMB = substantive-model-compatible multilevel MI (sequential modeling). The Monte Carlo error ranged from 0.1% to 1.5% (median 0.5%)

The results for the bias are summarized in Fig. 3. Similar to study 1, using FCS or LD (under MAR) led to biased estimates of the regression coefficients for the effect of *x* at level 1 and the CLI (*β*_1_ and *β*_4_) as well as the slope variance ($\tau _1^2$) in most of the simulated conditions. By contrast, Blimp and mdmb both provided essentially unbiased estimates of the parameters. In contrast to study 1, this also included the estimates of the slope variance ($\tau _1^2$), which were unbiased in all conditions. However, in contrast to Blimp and mdmb, the estimates obtained with jomo tended to be biased in most conditions, reflecting the fact that this method could not fully accommodate the substantive analysis model during MI.

The coverage rates for the 95% confidence intervals are shown in Table 4 for the regression coefficient of the CLI (*β*_4_). Overall, the results were similar to study 1 and followed the same pattern as the bias in most cases. Specifically, the coverage rates were generally close to the nominal level of 95% when Blimp or mdmb were used, whereas the coverage rates often dropped well below the nominal value when FCS or LD (under MAR) were used, especially in larger samples. However, although the estimates of the parameters of interest that jomo provided were sometimes biased, this did not appear to influence the coverage rates of the 95% confidence intervals, which were still close to the nominal level in most cases.

**Table 4:** Coverage of the 95% confidence intervals for the cross-level-interaction (CLI, *β*_4_) in conditions with moderate ICCs (*ρ*_*Ix*_ = *ρ*_*Iy*_ = .20) in study 2

		MCAR (*λ*_1_ = 0)						MAR (*λ*_1_ = .70)					
*n*	*J*	CD	LD	FCS	JOMO	BLIMP	MDMB	CD	LD	FCS	JOMO	BLIMP	MDMB
10	50	93.5	93.0	94.7	93.4	93.6	93.8	94.5	**89.3**	94.5	93.5	93.9	94.3
	100	95.3	94.1	**92.0**	94.6	94.2	94.5	95.0	**83.7**	**91.1**	95.1	94.6	94.7
	200	95.2	95.1	**91.6**	95.0	95.6	94.5	95.8	**77.5**	**86.7**	95.9	95.4	95.1
	500	95.6	94.9	**81.3**	94.6	94.1	95.1	95.3	**53.1**	**73.6**	96.1	95.5	96.2
	1000	95.5	95.5	**65.6**	94.5	95.6	95.4	94.5	**22.3**	**45.4**	94.7	93.4	94.4
20	50	95.2	93.9	93.7	94.2	94.1	94.7	94.5	**87.7**	93.4	95.1	94.8	95.0
	100	94.2	94.2	93.5	94.7	94.5	94.4	94.8	**79.3**	**88.5**	95.1	94.7	94.6
	200	95.9	95.3	**88.4**	95.3	95.1	94.9	94.1	**64.6**	**79.7**	96.4	94.7	94.7
	500	95.5	95.5	**76.8**	95.3	95.1	95.4	95.6	**35.1**	**63.6**	96.9	94.6	94.8
	1000	94.2	94.1	**62.4**	93.7	94.1	93.4	96.0	**7.3**	**34.6**	97.8	95.7	95.7

Note. Coverage rates below 92.5% are printed in bold. *n* = level 1 sample size; *J* = level 2 sample size; CD = complete data; LD = listwise deletion; FCS = multilevel MI (fully conditional specification); JOMO = substantive-model-compatible multilevel MI (joint modeling); BLIMP = substantive-model-compatible multilevel MI (fully conditional specification); MDMB = substantive-model-compatible multilevel MI (sequential modeling). The Monte Carlo error ranged from 0.2% to 1.2% (median 0.5%)

### Summary

The results of study 2 indicated that substantive-model-compatible methods for multilevel MI can provide unbiased estimates in multilevel analyses with interactions and random slopes even when the model includes centering to distinguish between the effects of explanatory variables at levels 1 and 2. By contrast, conventional methods for multilevel MI and LD were not able to preserve the relations between variables and led to biased estimates of the model parameters. However, in contrast to study 1, only Blimp and mdmb were able to provide unbiased results, whereas jomo was not able to fully accommodate the substantive analysis model. Nonetheless, it is important to emphasize that this does not constitute a restriction of the SMC-JM approach in general but only refers to specific implementations in software. More generally, this highlights the importance of the correct specification of the imputation model for ensuring unbiased estimates in multilevel analyses. In the previous simulations, we primarily investigated how nonlinear effects in the substantive analysis model can be accommodated with substantive-model-compatible methods for multilevel MI. In the following study, we extended our investigation to include cases with additional nonlinear associations between the explanatory variables.

## Study 3

### Data generation

In study 3, we aimed to investigate the effects of additional nonlinear associations between the variables, especially those that may exist between the explanatory variables *x* and *z*. For this reason, we simulated the explanatory variables in accordance with a nonlinear model. Specifically, *z* was standardized and followed a univariate normal distribution. Then, we generated *x* as follows:

13 $$ \renewcommand{\arraystretch}{0.8} \begin{array}{c} x_{j}^{L2} = \phi_{0} + \phi_{1} z_{j} + \phi_{2} {z_{j}^{2}} + \epsilon_{j}  , \quad \epsilon_{j} \sim N (0, \rho_{Ix}(1-R_{xz}^{2})) \\ x_{ij}^{L1} \sim N(0, 1-\rho_{Ix})  , \end{array} $$

where $R_{xz}^2$ denotes the total amount of variance explained in *x* by both the linear and nonlinear effects of *z*, and the regression coefficients *ϕ*_0_, *ϕ*_1_, and *ϕ*_2_ were chosen in such a way that *x* would have a mean of zero and the linear and nonlinear effects would contribute a given amount of variance (in %) to the total $R_{xz}^2$. Then, we simulated the outcome variable *y* in accordance with the following substantive analysis model:

14 $$ \begin{array}{@{}rcl@{}} y_{ij} & = & \beta_{0} + \beta_{1} x_{ij} + \beta_{2} z_{j} + \beta_{3} x_{ij} z_{j} + \beta_{4} x_{ij}^{2} + \beta_{5} {z_{j}^{2}}\\ && + u_{0j} + u_{1j} x_{ij} + e_{ij}  . \end{array} $$

This model is illustrated in Fig. 1 (panel C) and is similar to the data generating model in study 1 in that it includes only “overall” (i.e., conflated) effects of the explanatory variables at level 1. However, the model includes additional nonlinear (i.e., quadratic) effects of the explanatory variables *x* and *z*. The distributions of the random effects and residuals were the same as in study 1, and missing data were induced in *x* as before.

#### Simulated conditions

The simulated conditions are summarized in Table 1. To keep the design as simple as possible, we only simulated conditions with large samples at level 1 (*n* = 20) and level 2 (*J* = 1000). In addition, we fixed the total $R_{xz}^2$ to .50 and varied the relative weight of the nonlinear effect of *z* to the $R_{xz}^2$ in finer steps (*w* = 0, .25, .50, .75, 1), which reflected conditions in which the relation between *x* and *z* was linear or nonlinear to varying degrees (i.e., completely linear, mixed, or completely nonlinear). Note that, although this value for the total $R_{xz}^2$ may be considered large, it implies a reasonable range of the values of the correlation between *x* and *z*, which takes values roughly between 0 and .7, depending on the weight *w* of the nonlinear term (e.g., *ρ*_*xz*_ ≈ .7 if *w* = 0 and *ρ*_*xz*_ = 0 if *w* = 1). For the regression coefficients in the substantive analysis model, we chose a uniform value of .15. As before, each condition was replicated 1000 times.

### Procedures and parameters of interest

The procedures used for the treatment of missing data were the same as in studies 1 and 2. However, of the three methods for substantive-model-compatible multilevel MI, only the SMC-SM approach (mdmb) allowed us to take the nonlinear association between the explanatory variables into account. By contrast, the SMC-JM (jomo) and SMC-FCS (Blimp) approaches also accommodated the substantive analysis model but did not allow the nonlinear association between the explanatory variables to be included in the specification of the imputation model. For this reason, we expected that mdmb would outperform jomo and Blimp. The parameters of interest primarily included the regression coefficients in the substantive analysis model, that is, the coefficients of the linear and quadratic effects of *x* and *z* (*β*_1_, *β*_2_, *β*_4_, and *β*_5_) as well as their CLI (*β*_3_). For each procedure and parameter of interest, we calculated the bias, the RMSE, and the coverage rates of the 95% confidence intervals as before. However, in the interest of space, we focus on only the bias.

### Results

**Fig. 4:**
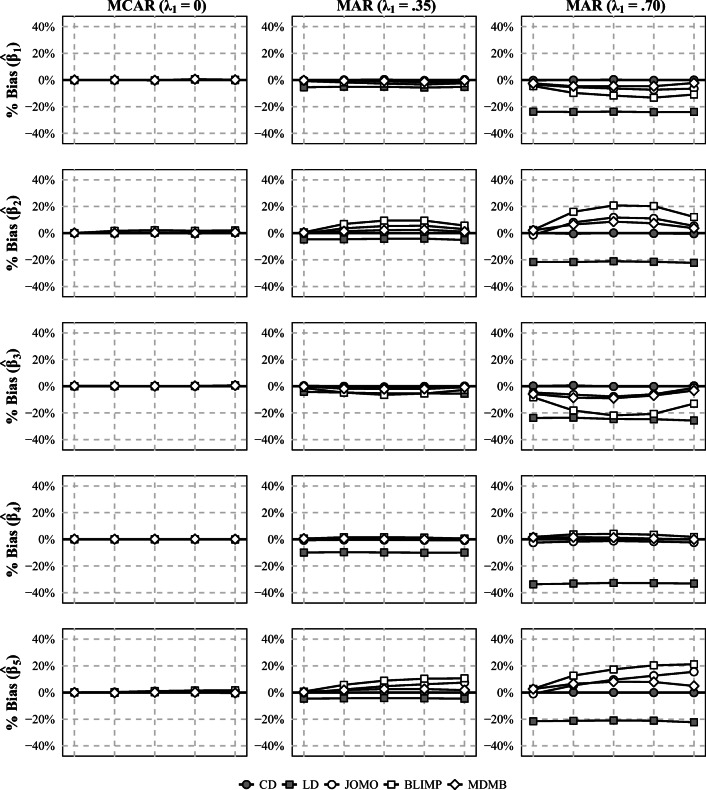
Bias (in %) of the estimated regression coefficients for the linear effect of *x* (*β*_1_), the linear effect of *z* (*β*_2_), the CLI (*β*_3_), the quadratic effect of *x* (*β*_4_), and the quadratic effect of *z* (*β*_5_) in study 3. *w* = relative weight of the nonlinear effect of *z* on *x*; CD = complete data; LD = listwise deletion; JOMO = substantive-model-compatible multilevel MI (joint modeling); BLIMP = substantive-model-compatible multilevel MI (fully conditional specification); MDMB = substantive-model-compatible multilevel MI (sequential modeling). The Monte Carlo error ranged from 0.1% to 0.4% (median 0.2%)

The results for the bias are summarized in Fig. 4. For simplicity, we did not include the results for FCS in this figure, because the bias that occurred with FCS often exceeded the bias that occurred when other methods were used by a substantial margin. Overall, the bias in the estimated parameters depended strongly on the missing data mechanism and the strength of the nonlinear association between the explanatory variables. Specifically, when the data were MCAR, all methods provided approximately unbiased results for all parameters of interest. Under MAR, all procedures sometimes produced biased results, but the amount of bias that was produced and the parameters affected by the bias often differed. Specifically, the estimates obtained with FCS and LD were usually the most biased regardless of the relative weight of the nonlinear association between the explanatory variables. By contrast, when the nonlinear association had no weight (*w* = 0), Blimp, jomo, and mdmb all provided reasonable parameter estimates with only little or no bias. When the nonlinear association had moderate weight (*w* = .25 to .75), jomo and mdmb still provided parameter estimates with relatively little bias (up to 12.6% for jomo and 9.0% for mdmb), whereas the bias obtained with Blimp tended to be larger (up to 21.9%). Finally, when the weight of the nonlinear association was large (*w* = 1), mdmb provided the estimates with the least amount of bias (up to 4.9%), whereas the bias obtained with Blimp and jomo was larger (up to 15.5% for jomo and 21.2% for Blimp).

### Summary

The results of study 3 illustrate two important points. First, in cases with systematically missing data (i.e., MAR), the treatment of missing data can be further complicated by the existence of additional nonlinear associations between the explanatory variables. In such a case, including additional nonlinear effects in the imputation model for the explanatory variables can be beneficial in the sense that it can reduce the bias in parameter estimates in multilevel analyses. Second, however, these differences tend occur in only a small number of cases, in which both the missing data mechanism and the nonlinear associations are relatively strong. For this reason, the potential benefits of including additional nonlinear effects in real-life applications (e.g., with SMC-SM) may be relatively small in comparison with the overall benefits of using substantive-model-compatible versus conventional methods for multilevel MI.

## Empirical example

In the following, we provide an illustration of the methods considered in this article using empirical data. The example is based on an example from Hoffman (2015, Chapter 8) and uses the data from the daily diary project in the MIDUS 2 study (Ryff & Almeida, 2017). The data include responses from 2022 adults (*M* = 56.2 years, range, 33–84 years) in a longitudinal study on the effects of daily (negative) mood and stress on perceived physical symptoms with ten measurements collected over 2 weeks. The data contain missing values in both negative mood (7.9%) and stress (7.9%). For ease of interpretation, we centered the data for all variables before the analysis. The computer code used to run the example analysis is provided in Supplement A of the online supplemental materials (https://osf.io/aeqd2).

The substantive analysis model for this example can be used to investigate the effects of negative mood and stress as well as the participants’ gender and age on physical symptoms. Specifically, the model includes effects of negative mood and stress both within (level 1) and between persons (level 2) as well as effects of gender and age across persons (level 2) and potential interactions between them. In the notation used by Raudenbush and Bryk (2002), the model can be written as follows. For person *j* at time point *i*,

### Level 1:

$$ \begin{array}{@{}rcl@{}} \textit{Symptoms}_{ij} &=& {} \beta_{0j} + \beta_{1j} (\textit{Mood}_{ij} - \overline{\textit{Mood}}_{\bullet j}) + \beta_{2j} \textit{Stress}_{ij}\\ &&+ \beta_{3} (\textit{Mood}_{ij} - \overline{\textit{Mood}}_{\bullet j}) \textit{Stress}_{ij} + e_{ij} \end{array} $$

### Level 2:

15 $$ \begin{array}{@{}rcl@{}} \beta_{0j} &= {} & \beta_{0} + \beta_{4} \overline{\textit{Mood}}_{\bullet j} + \beta_{5} \overline{\textit{Stress}}_{\bullet j} + \beta_{6} \textit{Gender}_{j}\\ &&+ \beta_{7} \textit{Age}_{j} + \beta_{8} \overline{\textit{Mood}}_{\bullet j} \overline{\textit{Stress}}_{\bullet j} \\ &&+\beta_{9} \overline{\textit{Mood}}_{\bullet j} \textit{Gender}_{j} + \beta_{10} \overline{\textit{Stress}}_{\bullet j} \textit{Gender}_{j}\\ &&+ \beta_{11} \textit{Gender}_{j} \textit{Age}_{j} + u_{0j} \\[1.0ex] \beta_{1j} &=& \beta_{1} + \beta_{12} \overline{\textit{Stress}}_{\bullet j} + \beta_{13} \textit{Gender}_{j} + u_{1j} \\[1.0ex] \beta_{2j} &=& \beta_{2} + \beta_{14} \overline{\textit{Mood}}_{\bullet j} + \beta_{15} \textit{Gender}_{j} \end{array} $$

At the within-person level (level 1), the model includes effects of negative mood and stress. In addition, the model includes a random slope for negative mood, which allows the within-person effect of negative mood to vary across persons. At the between-person level (level 2), the model includes effects of negative mood, stress, gender, and age. Finally, the model includes a number of interaction effects, both within (level 1) and between persons (level 2) as well as CLIs to investigate whether the within- and between-person effects of negative mood and stress are moderated by time-varying (level 1) or person-level characteristics (level 2). Notice that missing data occur in both of the two time-varying explanatory variables (negative mood and stress). In such a case, most software packages for multilevel analyses will remove cases with incomplete data, leading to a reduction in sample size. In the present example, a total of 1280 observations would be dropped from the analysis.

To handle the missing data, we used the substantive-model-compatible methods for multilevel MI as outlined above. This included SMC-JM, implemented in the R package jomo; SMC-FCS, implemented in the Blimp software; and SMC-SM, implemented in the R package mdmb.^5^

Using each method, we generated 20 imputations for the missing values. Finally, we used the R packages lme4 (Bates et al., 2019) and mitml (Grund et al., 2019) to analyze the imputed data sets and pool the results. For comparison, we also included LD.

The results are summarized in Table 5. In this example, negative mood was associated with perceived physical symptoms primarily at the person-level (level 2), indicating that participants with a more negative mood also experienced more physical symptoms. The within-person effect of negative mood was smaller but also positive. In addition, there seemed to be positive effects of the participants’ gender and age, indicating that female and older participants reported more physical symptoms. There were no main effects of stress. However, stress seemed to moderate the effects of negative mood both within and between persons as well as across levels, indicating that the relation between negative mood and physical symptoms is stronger for participants who experience more stressful events on a given day or on average. Overall, the results differed relatively little across the procedures, which usually provided estimates well within one unit of a standard error (SE). However, there were two exceptions. First, the CLI between stress (at level 1) and negative mood (at level 2) was slightly larger and just barely statistically significant with jomo and Blimp, whereas it was slightly smaller and not significant with mdmb. Second, the estimates of the slope variance differed across procedures, which was largest with Blimp, slightly lower with LD and jomo, and lowest with mdmb. However, these differences should not be overstated given that the model is fairly complex and the uncertainty with which the parameters were estimated was relatively high despite the low proportion of missing data.

**Table 5:** Results from the empirical example

	LD		JOMO		BLIMP		MDMB	
Parameter	Est.	*SE*	Est.	*SE*	Est.	*SE*	Est.	*SE*
Intercept (*β*_0_)	-0.048	0.054	-0.171**	0.053	-0.173**	0.053	-0.202***	0.052
*Level 1*								
Mood (*β*_1_)	0.576***	0.128	0.737***	0.138	0.715***	0.145	0.621***	0.138
Stress (*β*_2_)	-0.084*	0.042	-0.078	0.044	-0.074	0.045	-0.076	0.042
Mood × Stress (*β*_3_)	0.381**	0.144	0.470**	0.152	0.489**	0.166	0.428**	0.151
*Level 2*								
Mood (*β*_4_)	1.933***	0.223	1.568***	0.235	1.650***	0.233	1.579***	0.216
Stress (*β*_5_)	0.183	0.201	0.242	0.204	0.244	0.206	0.082	0.204
Gender (*β*_6_)	0.570***	0.069	0.559***	0.068	0.553***	0.068	0.548***	0.067
Age (*β*_7_)	0.014***	0.004	0.015***	0.004	0.015***	0.004	0.015***	0.004
Mood × Stress (*β*_8_)	0.780**	0.254	0.688**	0.257	0.711**	0.258	0.645*	0.253
Mood × Gender (*β*_9_)	-0.015	0.264	0.086	0.268	0.079	0.267	0.058	0.266
Stress × Gender (*β*_10_)	1.728***	0.393	1.941***	0.473	2.321***	0.479	1.604***	0.468
Gender × Age (*β*_11_)	-0.001	0.005	-0.000	0.005	-0.000	0.005	0.000	0.005
*Cross-level effects*^*a*^								
Mood × Stress (*β*_12_)	0.275*	0.136	0.422**	0.152	0.435**	0.164	0.408**	0.154
Mood × Gender (*β*_13_)	-0.075	0.056	-0.098	0.059	-0.085	0.061	-0.086	0.056
Stress × Mood (*β*_14_)	0.549*	0.262	0.611*	0.308	0.724*	0.323	0.481	0.305
Stress × Gender (*β*_15_)	0.196	0.116	0.297*	0.139	0.114	0.138	-0.130	0.108
*Variance components*								
Intercept ($\tau _0^2$)	1.535		1.390		1.396		1.398	
Slope (Mood, $\tau _1^2$)	1.535		1.399		1.801		1.106	
Residual (*σ*^2^)	1.659		1.797		1.785		1.823	

^∗^*p* < .05, ^∗∗^*p* < .01, ^∗∗∗^*p* < .001 (two-sided)

^*a*^ For the cross-level effects, level 1 variables are named first (before the “×”) and level 2 variables second (after the “×”)

Note. LD = listwise deletion; JOMO = substantive-model-compatible multilevel MI (joint modeling); BLIMP = substantive-model-compatible multilevel MI (fully conditional specification); MDMB = substantive-model-compatible multilevel MI (sequential modeling)

## Discussion

The analysis of multilevel models is often complicated by the presence of missing data. It has been pointed out by several authors that incomplete predictor variables in multilevel models with cross-level interactions and nonlinear effects are currently not easy to handle in mainstream multilevel software (Grund et al., 2018b; Enders et al., 2018). In the present paper, we introduced a sequential modeling approach to multilevel MI (the SMC-SM approach), which is implemented in the R package mdmb and allows for a substantive-model-compatible imputation of missing data in multilevel models with random slopes and nonlinear effects. We showed that this approach provides a versatile and effective tool for treating missing data in multilevel analyses. In the following, we discuss the limitations of our study and the sequential modeling approach and consider additional applications, in which the sequential modeling approach can be used.

In the SMC-SM approach, it is important to recognize that its performance—as with MI in general—depends on the correct specification of the imputation models. Specifically, the order of the conditional models used to impute the explanatory variables must be explicitly specified. In other words, it must be decided which variables occur “early” vs. “late” in the sequence, where “early” refers to the variables which are assigned with the smallest conditional models with the fewest predictors. For practical applications, researchers have proposed several strategies that allow the order of the conditional models to be selected in such a way that problems with misspecified imputation models can be greatly reduced (Ibrahim et al., 2005; Murray, 2018). First, they recommend that the sequence begin with the conditional models for level 2 variables, followed by level 1 variables. Second, at levels 1 and 2, the variables can be ordered by type, beginning the sequence with categorical variables followed by continuous variables. These two strategies are beneficial because models for categorical or level 2 variables are more difficult to estimate than models for continuous or level 1 variables. Third, the variables should be ordered by their percentage of missing data, beginning the sequence with the variables that exhibit a lower percentage of missing values. This is motivated by the fact that the specification of a conditional model has a smaller influence on the missing data treatment if variables with fewer missing values are placed earlier in the sequence because the unexplained variation tends to be larger for the variables that come early in the sequence.

One particular challenge with substantive-model-compatible MI occurs if there is interest in more than one substantive analysis model, for example, in model selection or research on multiple outcomes. In such a case, it can be required to run the imputation procedure multiple times with different specifications of the substantive analysis model. However, there are several exceptions. First, model selection can be performed on a single set of imputations as long as the models under consideration are nested in each other, in which case the imputation model should be specified in accordance with the most general model. Second, when analyzing multiple outcomes, a single set of imputations can often be used when all models share the same form and include a common set of explanatory variables. In this case, in SMC-SM, the sequence of models can be extended to accommodate multiple substantive analysis models.

Our study considered only cases in which the data were MAR (or MCAR). In practice, this assumption may be violated, and the data may be missing *not* at random (MNAR) even after auxiliary variables are taken into consideration in the treatment of missing data. Stubbendick and Ibrahim (2003) showed how the SMC-SM approach can be used to impute incomplete multilevel data under MNAR by adding a statistical model for the occurrence of missing values in the predictor variables (i.e., missingness indicators). However, for MNAR data, strong assumptions about the missing data mechanism are required to estimate models for the missingness indicators. More specifically, for each missingness indicator, a subset of variables (in the imputation model) that cause the missingness in that variable must be identified. Alternatively, it has been argued that models for MNAR data should primarily be used to conduct sensitivity analyses that allow researchers to investigate the extent to which the results are robust to the MAR assumption in comparison with different MNAR mechanisms (Carpenter & Kenward, 2013).

In the present article, we focused on the SMC-SM approach for two-level data. However, the sequential modeling approach can be used for multilevel data with an arbitrary number of levels, both hierarchical (e.g., three-level data) and nonhierarchical (e.g., cross-classified data; see Rasbash & Browne 2008), and with explanatory variables and the outcome variable located at any level in the multilevel structure. These extensions have also been implemented in the mdmb package. However, research on the performance of MI in data with three or more levels is still scarce, and it would be an important topic for future research to investigate under which conditions (e.g., sample size, intraclass correlations) multilevel MI provides unbiased and efficient estimates. In this context, it is also important to point out that the sequential modeling approach for multilevel data can also be implemented and used to generate imputations with general-purpose Bayesian software packages such as BUGS and JAGS (see also Erler et al., 2017; Grund et al., 2018b). However, implementing BUGS or JAGS code for the SMC-SM approach is often not a viable option for researchers who do not have advanced experience with these software packages, in which case specific implementations of these methods can be helpful (for packages implementing the SMC-SM approach using existing software for Bayesian analysis, see also Erler et al. 2019).

For multilevel analyses without random slopes or nonlinear effects, conventional methods for multilevel MI include the FCS approach as well as joint modeling (JM), which is available in many statistical software packages (e.g., in M*plus*, MLwiN, or the R packages jomo and pan; Schafer & Yucel 2002b). Several extensions of the JM approach have also been considered as promising alternatives for handling missing data in models with random slopes or interaction effects. For example, multilevel JM has been extended to include random covariance matrices at level 1 (Yucel, 2011) and this method has been considered an alternative for handling missing data in models with random slopes (e.g., Enders et al., 2018; Quartagno & Carpenter 2016). In addition, implementations of multilevel JM based on latent class analysis or mixture distributions could provide a promising alternative to substantive-model-compatible multilevel MI in applications with complex interactions between multiple variables (e.g., Vidotto et al., 2018). These methods should be considered further in future research.

In summary, our article showed that a sequential modeling approach has great potential for handling missing data in complex multilevel models that include nonlinear effects. We introduced the R package mdmb, which facilitates the application of the SMC-SM approach and provides a flexible way to treat missing data in multilevel analyses. However, more generally, our article also demonstrated the utility and effectiveness of substantive-model-compatible methods in comparison with conventional methods for multilevel MI. Software implementations for substantive-model-compatible multilevel MI such as those in mdmb, jomo, Blimp, and others provide user-friendly implementations of these methods to a wide audience, and we hope that this article will help to further their application in research practice.

## Footnotes

1 In the literature, acceptance rates of roughly between .40 and .50 are considered optimal (Gelman et al., 2014; Hoff, 2009) for obtaining an MCMC chain that has a relatively low autocorrelation and mixes well. To achieve a desirable acceptance rate, the standard deviation of the proposal distribution is tuned during the burn-in phase of the MCMC algorithm by applying an adaptive procedure described by Draper (2008, p. 116).

2 Starting with version 3, the Blimp software also supports SMC-SM in addition to SMC-FCS.

3 In the present case, where only *x* is incomplete, “passive” imputation is not strictly needed. Instead, the product term of *y* and *z* used in the “reversed” imputation model can simply be calculated a priori. However, in the more general case with missing data in more than one variable, “passive” imputation is needed to update product terms to accommodate the most recently imputed values.

4 For all methods, we placed an improper (uniform) prior on the fixed effects and an inverse Wishart prior, *W*^− 1^(*ν*,**Σ**), on the covariance matrices at levels 1 and 2 with different specifications of the degrees of freedom and the scale matrix. For jomo, these were specified as least informative priors (*ν* = *k*, **Σ** = **I**_*k*_ for a matrix of size *k*; see Schafer & Yucel 2002b). For Blimp and mdmb, we used improper priors (*ν* = −(*k* + 1), **Σ** = 0 ⋅**I**_*k*_).

5 Notice that the substantive analysis model uses centering to distinguish between the within- and between-person effects of negative mood. These effects could be accommodated by Blimp and mdmb, whereas jomo included only the “overall” (i.e., conflated) effect of negative mood.
